# Deciphering the response and resistance to immune-checkpoint inhibitors in lung cancer with artificial intelligence-based analysis: when PIONeeR meets QUANTIC

**DOI:** 10.1038/s41416-020-0918-3

**Published:** 2020-06-16

**Authors:** Joseph Ciccolini, Sébastien Benzekry, Fabrice Barlesi

**Affiliations:** 1 Centre de Recherche en Cancérologie de Marseille, SMARTc, Aix-Marseille Université, Inserm U1068, CNRS UMR 7258, Institut Paoli Calmettes, 13009 Marseille, France; 2 Assistance Publique Hôpitaux de Marseille, Marseille, France; 3 MONC Team, Institut National de Recherche en Informatique Appliquée Bordeaux Sud Ouest and Institut Mathématique de Bordeaux, CNRS UMR 5251, Bordeaux, France; 4 Centre de Recherche en Cancérologie de Marseille, Aix-Marseille Université, Inserm U1068, CNRS UMR 7258, Institut Paoli Calmettes, 13009 Marseille, France; 5 Gustave Roussy Cancer Campus, Villejuif, France

**Keywords:** Cancer immunotherapy, Computational models

## Abstract

This project aims to generate dense longitudinal data in lung cancer patients undergoing anti-PD1/PDL1 therapy. Mathematical modelling with mechanistic learning algorithms will help decipher the mechanisms underlying the response or resistance to immunotherapy. A better understanding of these mechanisms should help identifying actionable items to increase the efficacy of immune-checkpoint inhibitors.

## Main

The era of precision medicine mostly relies on identifying biomarkers likely to help oncologists forecasting clinical outcome. Immune-checkpoint inhibitors (ICIs) are game-changing drugs in oncology, as some cancers with once-dismal prognosis now show survival rates that count in years. Non-small-cell lung cancer (NSCLC) is typically a disease, in which the clinical outcome has been dramatically improved over a short period of time with first targeted therapies and then the successive approvals of anti-programmed death 1 (PD1) and anti-programmed death ligand 1 (PDL1) biologicals.^1^ However, after the initial frenzy ignited by promising Phase 3 trials with nivolumab, pembrolizumab or more recently atezolizumab, alone or in combination with standard chemotherapy, many questions remain unsolved. In particular, the lack of robust and fully validated biomarkers for predicting response to ICIs remains a major issue. PDL1 expression, tumour mutational burden, initial tumour size, history of steroids or antibiotics use, MSI status and gut microbiota characteristics have all been pointed independently as possible factors impacting on the response or survival in patients treated next with ICIs.^2–4^ However, many uncertainties remain, essentially because of possible confounding factors, and the lack of global understanding of the utterly complex interplay between drugs, immune system, tumour cells and tumour microenvironment plus all physiopathological parameters and patients’ characteristics likely to interfere with harnessing tumour immunity.^5^ Consequently, in many respects, treating patients with immunotherapy looks like a big lottery—the winning tickets (i.e., response or remission with no or little immune-related toxicities) being apparently distributed randomly among patients.^6^ In this context, the association between clinical investigators, biologists, mathematicians and industrialists is crucial to accurately exploit big clinical and biomarker data, and stratify patients prior to therapeutic decision. The PIONeeR project is built upon a large biomarker programme, and a randomised, umbrella clinical trial aiming at understanding, predicting and overcoming resistance to PD1 and PDL1 ICIs. The study (NCT03493581) first investigates a wide panel of putative tissue and liquid biomarkers aiming at deciphering the immune contexture in 450 advanced lung cancer patients treated with nivolumab, pembrolizumab or atezolizumab alone or in combination with chemotherapy. Tested biomarkers include advanced immunohistochemistry coupled to digital pathology analyses, such as CD8^+^/PDL1^+^ co-localisation (Immunoscore®), or complex immune cell population localisation and quantification, including myeloid-derived suppressor cells, blood immune monitoring, including rare cell subsets, genomics and transcriptomics, gut microbiota exploration, study of vascular factors, pharmacokinetics and PK/PD modelling. Both progressing and responding patients are closely monitored so as to better uncover unbiased predictive biomarkers. Patients with progressive disease before 24 weeks of treatment will be next further randomised in a second clinical step testing at least three combinatorial regimens of targeted therapies with the anti-PDL1 durvalumab with full longitudinal monitoring as well. The primary objective is to highlight immune algorithms predicting anti-PD1/PDL1 primary and adaptive resistance to stratify patients prior to ICI treatment. Importantly, all the collected data will be used next as part of the QUANTIC add-on project, which is an original collaboration between the French National Institute for Research on Computer Science and Applied Mathematics (better known as INRIA) and the PIONeeR consortium. The primary objective of QUANTIC is to develop and validate a mechanistic, dynamic model of response and resistance to immune-checkpoint inhibition, leveraging the unique, large scale, multi-modal and longitudinal data collected during the PIONeeR clinical study. Indeed, artificial intelligence techniques are required to analyse the ‘big data' generated by PIONeeR (e.g., immunomonitoring alone will result in hundreds of quantitative variables per time point, per patient), plus additional APHM routine patients (i.e., 521 NSCLC patients who have received anti-PD1/PDL1 treatment over the last 5 years). On the other hand, mechanistic modelling consists of designing physiologically based mathematical constructs for the systemic kinetics of the disease and its response to ICIs. Such models have superior value to artificial intelligence algorithms because they are interpretable. This allows them to account for the biological meaning of part of the data (e.g., quantification of immune players), and to test biological hypotheses, which improves our mechanistic understanding of the processes at play. However, the fact that not all the data have a biological meaning, combined to the large number of variables in some data modalities (e.g., genomics or immune monitoring), as well as the requirement for non-linear covariate models, are all rationales to keep parts of the modelling biologically agnostic, i.e., relying on machine learning alone. As stated above, the major clinical challenge with immunotherapy today is the wide inter-individual heterogeneity in response to ICIs. To address this issue and quantify this variability, mixed-effect statistical learning will be used for the first time. All patients’ data will be pooled together for the learning process, which strengthens estimation of the mechanistic parameters. Machine learning for inclusion of baseline covariates will further yield new algorithms able to predict the response/relapse patterns, including possible pseudo- or hyper-progression. Finally, model parameters and longitudinal analysis will be used to predict overall survival. This unique and entire multi-modal framework for mechanistic description and prediction of longitudinal kinetics of hundreds of coupled biomarkers will go much beyond the current state of the art in clinical quantitative modelling since most of the current studies model only the sum of the longest diameters from RECIST target lesions as readouts.^7^

Overall, the PIONeeR and QUANTIC projects highlight how state-of-the-art computational oncology, biomarker-based investigations and clinical trials should join their forces for deciphering the complex mechanisms explaining the variability in clinical outcomes with immunotherapy.^8^ As such, it should bring substantial progress for the in-depth understanding of resistance to ICIs in advanced lung cancer patients. The final mathematical models will be used in the future as a new powerful tool for decision-making, i.e., by clustering patients prior to the start of immunotherapy through a unique combination of somatic and germinal traits. In addition, depending on the actionable items that will have emerged from the project (e.g., drug exposure parameters calling for adaptive dosing strategies), patients once doomed to progress upon immunotherapy will benefit from customised treatment so as to increase their odds of success.

## Data Availability

Not applicable.
